# Handling Complex Missing Data Using Random Forest Approach for an Air Quality Monitoring Dataset: A Case Study of Kuwait Environmental Data (2012 to 2018)

**DOI:** 10.3390/ijerph18031333

**Published:** 2021-02-02

**Authors:** Ahmad R. Alsaber, Jiazhu Pan, Adeeba Al-Hurban

**Affiliations:** 1 Department of Mathematics and Statistics, University of Strathclyde, Glasgow G1 1XH, UK; jiazhu.pan@strath.ac.uk; 2 Department of Earth and Environmental Sciences, Faculty of Science, Kuwait University, P.O. Box 5969, Safat 13060, Kuwait; Q8geo@hotmail.com

**Keywords:** missing imputation, random forest, high dimensional data, missing data mechanism, air quality

## Abstract

In environmental research, missing data are often a challenge for statistical modeling. This paper addressed some advanced techniques to deal with missing values in a data set measuring air quality using a multiple imputation (MI) approach. MCAR, MAR, and NMAR missing data techniques are applied to the data set. Five missing data levels are considered: 5%, 10%, 20%, 30%, and 40%. The imputation method used in this paper is an iterative imputation method, missForest, which is related to the random forest approach. Air quality data sets were gathered from five monitoring stations in Kuwait, aggregated to a daily basis. Logarithm transformation was carried out for all pollutant data, in order to normalize their distributions and to minimize skewness. We found high levels of missing values for $NO_{2}$ (18.4%), CO (18.5%), $PM_{10}$ (57.4%), $SO_{2}$ (19.0%), and $O_{3}$ (18.2%) data. Climatological data (i.e., air temperature, relative humidity, wind direction, and wind speed) were used as control variables for better estimation. The results show that the MAR technique had the lowest RMSE and MAE. We conclude that MI using the missForest approach has a high level of accuracy in estimating missing values. MissForest had the lowest imputation error (RMSE and MAE) among the other imputation methods and, thus, can be considered to be appropriate for analyzing air quality data.

## 1. Introduction

Air quality monitoring is conducted with the aim of protecting public health. Numerous air contaminants have been found to have harmful effects on human health. The air quality in cities varies, due to concentrations of particulate matter 10 micrometers ($PM_{10}$), nitrogen dioxide ($NO_{2}$), ozone ($O_{3}$), carbon monoxide (CO), and sulfur dioxide ($SO_{2}$), from emission sources including vehicle exhaust, manufacturing operations, and chemical facilities, among other sources.

A major challenge in air quality data management is determining how to deal with missing data values. Missing information in data sets occurs for multiple reasons, such as impaired equipment, insufficient sampling frequency, hardware problems, and human error [1]. Incomplete data sets affect the applicability of specific analyses, such as receptor modeling, which generally requires a complete data matrix [2]. The occurrence of missing data, no matter how infrequent, can bias findings on the relationships between air contaminants and health outcomes [3]. Incomplete data matrices may provide outcomes that vary significantly, compared to the results from complete data sets [4].

To gain a more complete data set, researchers must decide whether to discard or impute (i.e., substitute for) missing data. Ignoring missing values is typically not warranted, as valuable information is lost, which may compromise inferential power [5]. Therefore, the most appropriate option is to impute the missing data. Yet, the systematic differences between real and substituted data can also lead to unwanted bias. Therefore, it is vital to determine an optimal approach for estimating missing values. Several problems have been linked with missing data [6]. These challenges include statistical power reduction, bias as a result of inconsistent data, difficulties in managing the data during statistical analyses, and low efficiency. The criteria implemented for measures to deal with missing data in time-series analysis rely on the missing data replacement mechanism and missing data pattern [7]. Such challenges are especially problematic when the missing data exceed 60 percent, where existing methods have significant difficulty in addressing such situations [8].

This study focuses on a case study of missing data related to air quality monitoring. The Kuwait environmental public authority (KEPA) is mandated with the responsibility for measuring air quality. A data set collected from five fixed monitoring stations was associated with missing data, likely caused by multiple reasons. One is that there were a large number of routine maintenance changes in the monitoring sites. Second, simple human error occurred. Third, there were some tagging problems that necessitated the exclusion of some data.

The main purpose of this paper was to find the best imputation method to estimate the missing values for the measured pollutants ($SO_{2}$, $NO_{2}$, CO, $O_{3}$, and $PM_{10}$) in the KEPA data sets. The imputation methods used in this paper are: multivariate imputation by chained equations using random forest (RF), k-nearest neighbor (kNN), Bayesian principal component analysis (BPCA), multiple imputation using expectation maximization with bootstrapping (EM with Bootstrapping), predictive mean matching (PMM), and the proposed iterative imputation method (missForest) based on a random forest. Two tests, root mean square error (RMSE) and mean absolute error (MAE), are used to compare the performances of the imputation methods. For the error indicators (RMSE or MAE), the larger the value, the greater the error. The end product is an outline of the best approaches for managing missing data in a data set that is critical for public health in Kuwait.

It is important to describe the factors that may lead to missing data in statistical analyses. The first instance of missing data is missing completely at random (MCAR), whereby the missing data result from either the observer not collecting the necessary information or the reporting of incomplete or false information. The second instance of missing data is missing at random (MAR), whereby the extent of data missing depends on the type of data under observation. MAR is recommended when the missing data can be partially retrieved, depending on the existence of information related to the variables in the same data set. The third instance is missing not at random (MNAR), whereby the missing data are dependent on the actual values absent for statistical analysis. Among the three types of missing data in statistical analysis, MAR and MNAR are the most common [9]. When the type of missing data tends towards MAR, multiple imputation techniques are more suitable than other techniques, such as listwise deletion [10].

### 1.1. Missing Completely at Random (MCAR)

For MCAR, the chance of missing data values is the same across all instances. It can be interpreted as the cause of missing data values not being related to the data collected. For instance, a random sample of a population, whereby each individual from the population has an equal chance of being selected for the sample. This would mean that not all members of the population were present among the selected sample. Therefore, the data and values of the members not selected would be missing from the statistical analysis. The following example describes an instance in which MCAR occurs in statistical analysis:

Suppose that *Y* is an n×p matrix which includes all *p* variables with *n* cases in the sample. Let the observed values be denoted as $\left(Y_{\mathrm{obs}}\right)$, while the missing values are denoted as $\left(Y_{\mathrm{mis}}\right)$. The matrix *R* spots the missing values locations in *Y*. The observations of *R* and *Y* are denoted as $r_{\mathrm{ij}}$ and $y_{\mathrm{ij}}$, respectively. Thus, $r_{\mathrm{ij}}=1$ when $y_{\mathrm{ij}}$ is observed, while $r_{\mathrm{ij}}=0$ when $y_{\mathrm{ij}}$ is missing. Then, the distribution of *R* depends upon $Y=\left(Y_{\mathrm{obs}},Y_{\mathrm{mis}}\right)$. We can write $\mathrm{Pr}\left(R|Y_{\mathrm{obs}},Y_{\mathrm{mis}},\Psi \right)$ when the data are said to be assumed as MCAR, if:

(1) $$\mathrm{Pr}\left(R=0|Y_{\mathrm{obs}},Y_{\mathrm{mis}},\Psi \right)=\mathrm{Pr}\left(R=0|\Psi \right),$$

where ψ consists of the parameters of the missing data in the model. This means that the probability of missing a data value depends only on the estimated parameters in the model.

### 1.2. Missing at Random (MAR)

For MAR, the chance of data values missing is equal across all categories. MAR is, therefore, a more diverse instance, compared to MCAR; for instance, when selecting a sample from a population based on certain characteristics, the resulting missing data can be categorized as MAR. Statistical software for multiple imputations usually assumes that the data are MAR [11]. Therefore, the probability of data missing is dependent on the data under observation:

(2) $$\mathrm{Pr}\left(R=0|Y_{\mathrm{obs},}Y_{\mathrm{mis}},\Psi \right)=\mathrm{Pr}\left(R=0|Y_{\mathrm{obs}},\psi \right).$$

The KEPA data are best classified as MAR.

### 1.3. Missing Not at Random (MNAR)

For MNAR, the chance of data not being available is dependent on reasons unknown to the researcher. For instance, when conducting research, some respondents may decide to withhold information for reasons unknown to the researcher. Due to the nature of MNAR, it is often regarded as a more complex case in statistical analysis. It can be addressed by targeting some of the reasons respondents would choose to with hold information, $Y_{\mathrm{mis}}$, itself. It is represented:

(3) $$\mathrm{Pr}\left(R=0|Y_{\mathrm{obs}},Y_{\mathrm{mis}},\Psi \right).$$

The data set extracted from KEPA has extensive missing values. The missing data could have been due to routine maintenance, changes in the siting of monitors, human error, or tagging problems.

### 1.4. Ignoring the Missing Data Mechanism

One of the major issues that arise when performing imputations is whether the missing data come from the same distribution as the observed data ($Y_{\mathrm{obs}}$). As mentioned above, the observed data are made up of $Y_{\mathrm{obs}}$ and *R* with the joint density function $f\left(Y_{\mathrm{obs}},R|\theta ,\psi \right)$, which depends on the model estimated parameters θ for *Y*.

We can estimate θ without knowing ψ by defining the probability density function of the joint distribution of $Y_{\mathrm{obs}}$ and $Y_{\mathrm{mis}}$ as $f\left(Y|\theta \right)\equiv f\left(Y_{\mathrm{obs}},Y_{\mathrm{mis}}|\theta \right)$. Therefore, in order to compute the marginal probability density of $Y_{\mathrm{obs}}$, we integrate the missing data as:

(4) $$f\left(Y_{\mathrm{obs}}|\theta \right)=\int f\left(Y_{\mathrm{obs}},Y_{\mathrm{mis}}|\theta \right)dY_{\mathrm{mis}},$$

where the likelihood function of θ, according to $Y_{\mathrm{obs}}$ while ignoring the missing data, can be defined as:

(5) $$L_{ign}\left(\theta |Y_{\mathrm{obs}}\right)\propto f\left(Y_{\mathrm{obs}}|\theta \right).$$

Obtaining maximum likelihood (ML) estimates of θ can be done by maximizing the provided θ.

To build a more general model, we include *R* and specify the joint density distribution of *Y* and *R* as:

(6) f(Y,R|θ,ψ)=f(Y|θ)f(R|Y,ψ).

We can find the distribution of the observed data by integrating $Y_{\mathrm{mis}}$ from the joint density using θ and ψ, defined as:

(7) $$f\left(Y_{\mathrm{obs}},R|\theta ,\psi \right)=\int f\left(Y_{\mathrm{obs}},Y_{\mathrm{mis}}|\theta \right)f\left(R|Y_{\mathrm{obs}},Y_{\mathrm{mis}},\psi \right)dY_{\mathrm{mis}}.$$

Now, we can rewrite Equation (7) as:

(8) $$f\left(Y_{\mathrm{obs}},R|\theta ,\psi \right)=f\left(R|Y_{\mathrm{obs}},\psi \right)\int f\left(Y_{\mathrm{obs}},Y_{\mathrm{mis}}|\theta \right)dY_{\mathrm{mis}}=f\left(R|Y_{\mathrm{obs}},\psi \right)f\left(Y_{\mathrm{obs}}|\theta \right).$$

The missing data mechanism is ignorable for likelihood inference if

1. MAR: when the missing data pattern is missing at random; and
2. Distinctness: when the joint parameter space of (θ,ψ) is equal to the product of the parameter space of θ and ψ [12].

### 1.5. Multiple Imputation (MI)

Studies have shown that MI is unbiased if the missing rate for a variable exceeds 50% of the total missing values [13,14,15]. Researchers have debated the role of listwise deletion when solving for such missing data. Most research studies have concluded that, although the listwise deletion technique is not commonly used, it is applicable in some instances [16,17]. According to Marshall et al. [15], multiple imputation is favorable for computing missing data and especially applicable when the missing data rate is above 10% [18]. For instance, in a regression model, including the number of variables with a low rate of missing data. In such an instance, this may result in a rate of missing data that is higher in the full regression model, when compared to the outcomes of simple bivariant regressions. Therefore, it is critical for analysts to evaluate the total missing rate, as well as the partial missing one.

One limitation of applying a single imputation approach is that formulas of standard variance applied to filled-in data tend to underestimate the variance of the estimates; therefore, multiple imputation methods have been proposed [11]. The first step in such a method is specifying the single encompassing multivariate approach for all data sets. There are four types of multivariate models of data completion to consider [12]: (i) standard models, which impute under multivariate normal distributions; (ii) log-linear models, that have been used traditionally by social scientists in describing the associations among cross-classified data variables; (iii) general location models, which combine the log-linear approach for the variables that are definite with the multivariate model of standard regression for the continuous variables; and (iv) a two-level model of linear regression, which is mostly applied to multi-level data. The imputation model should be able to match the subsequent analysis and should be able to preserve the interactions of variables, which relates to the central point of the investigation discussed later in this paper.

A multiple imputation method balances ease of application and the quality of obtained results. The various imputations identify random errors that are appropriate to the process of imputation, making it possible to obtain unbiased estimates in all parameters. No deterministic method of imputation can achieve the same result. The technique also allows for departure from normality assumptions, while providing results that are adequate with low sample sizes or when significant amounts of data are missing.

Some requirements are necessary, in order to attain the desired results of multiple imputation [19]. First, there should be random data missing (MAR), which means that there is a dependence on observed variables and not missing observations. Second, the method of generating the values imputed should suit the analysis that subsequently follows. This maintains the associations between variables, which is a focus in the analysis shown later in this paper. Third, the model for imputation should coincide and agree with that of the investigation. Rubin has given a thorough description of these conditions. A remaining question, however, relates to adopting the most suitable practices for performing the imputations [20]. It is essential to have an awareness of the possible prediction problems, in order to reduce or minimize systematic error.

There have been many applications of multiple imputation in health, environmental [21,22], and industrial [23,24] data bases, as well as for survey data [25,26] and data mining approaches, which extract patterns from large data sets through a combination of artificial intelligence and statistical methods, that can be used for database management [23].

## 2. Materials and Methods

### 2.1. Multiple Imputation Using Random Forest Method

Let us assume that $X=\left(X_{1},X_{2},\ldots ,X_{p}\right)$ is a n×p-dimensional data matrix. We propose the use of the random forest technique for imputing missing observations. The random forest algorithm has a built-in routine to handle the values that are missing by weighing the frequency of values with the proximity of a random forest after the training of an initially imputed mean data set [27]. This approach requires a response variable that is complete and useful for forest training. Instead, we estimate the values of all the missing values directly, by use of a random forest that is trained on the observed data set, where *X* is the matrix of the complete data. $X_{s}$ contains all missing values at entries $i_{\mathrm{mis}}^{\left(s\right)}\subseteq \left\{1,\ldots ,n\right\}$. The data set can be separated into four parts:

1. $y_{\mathrm{obs}}^{\left(s\right)}$: the observed values of $X_{s}$.
2. $y_{\mathrm{mis}}^{\left(s\right)}$: the missing values of $X_{s}$.
3. $x_{\mathrm{obs}}^{\left(s\right)}$: the observations, $i_{\mathrm{obs}}^{\left(s\right)}=\left\{1,\ldots ,n\right\}\setminus i_{\mathrm{mis}}^{\left(s\right)},$ that belong in the other variables $X_{s}$.
4. $x_{\mathrm{mis}}^{\left(s\right)}$: the observations, $i_{\mathrm{mis}}^{\left(s\right)}$, that belong in the other variables $X_{s}$.

Note that $X_{\mathrm{obs}}^{\left(s\right)}$ and $X_{\mathrm{mis}}^{\left(s\right)}$ are not completely observed, as the index $i_{\mathrm{obs}}^{\left(s\right)}$ corresponds to the observed values of the variable $X_{s}$.

According to [28], the process starts with an initial guess for the missing values in X using a mean imputation approach or any other imputation method, depending on the data. Then, we sort the predictors $X_{s},s=1,\ldots ,p$, ascending or descending, $X_{s},s=1,\ldots ,p$, according to the number of missing values. Then, for each variable $X_{s}$, the missing values are imputed by random forest (i.e., the first fitting) with response $y_{\mathrm{obs}}^{\left(s\right)}$ and predictors $X_{\mathrm{obs}}^{\left(s\right)}$. Next, the missing values $y_{\mathrm{mis}}^{\left(s\right)}$ are estimated by applying the trained random forest to $X_{\mathrm{mis}}^{\left(s\right)}$. The imputation approach should be repeated until a stopping criterion is reached. Pseudo Algorithm 1 shows a representation of the missForest method (see Algorithm 1).

The stopping criterion (γ) is met when the difference between the last imputed data matrix and the previous one increases for the first time, with respect to both variable types. Here, the difference for the set of continuous variables N is defined as:

(9) $$\Delta_{N}=\frac{\sum_{j\in N}{\left(X_{new}^{imp}-X_{old}^{imp}\right)}^{2}}{\sum_{j\in N}{\left(X_{new}^{imp}\right)}^{2}},$$

and that for the set of categorical variables F as:

(10) $$\Delta_{F}=\frac{\sum_{j\in F}\sum_{i=1}^{n}I_{X_{new}^{imp}\neq X_{old}^{imp}}}{\#NA}.$$

Let X be an n×p matrix; set the stopping criterion (γ); set the initial guess for missing values. k← vector of sorted indices of columns in X w.r.t. increasing amount of missing values. $X_{old}^{imp}\leftarrow$ stores the previously imputed matrix. Fit a random forest: $y_{\mathrm{obs}}^{\left(s\right)}∼x_{\mathrm{obs}}^{\left(s\right)}$. Predict $y_{\mathrm{mis}}^{\left(s\right)}$ using $x_{\mathrm{mis}}^{\left(s\right)}$; $X_{new}^{imp}\leftarrow$ update the imputed matrix using the predicted $y_{\mathrm{mis}}^{\left(s\right)}$. Update γ and the imputed matrix $X^{imp}$. Where #NA is the number of missing values in the categorical variables F.

After imputing the missing values, the performance is assessed using the normalized root mean squared error [29] for the continuous variables, defined by:

(11) $$\mathrm{NRMSE}=\sqrt{\frac{\;\mathrm{mean}\;\left({\left(X^{\mathrm{true}}-X^{\mathrm{imp}}\right)}^{2}\right)}{\mathrm{var}\left(X^{\mathrm{true}}\right)}},$$

where $X^{\mathrm{true}}$ and $X^{\mathrm{imp}}$ are the complete data matrix and the imputed data matrix, respectively. In this study, all predictors are classified as continuous observations. The mean and variance are used as a short notation for empirical mean and variance computed over the missing values only.

When an RF is fit to the part that is observed on a variable, we use the out-of-bag (OOB) estimate of an error for the variable. When we meet the stopping criterion (γ), we average it over the variable set of that type, in order to obtain an approximation of the actual errors of imputation. We assess the performance of this estimate by comparing the absolute difference between the OOB imputation error estimate in all simulation runs and the true imputation error.

### 2.2. Process of Multiple Imputations (MI) Using Rubin’s Rules

**Figure 1:**
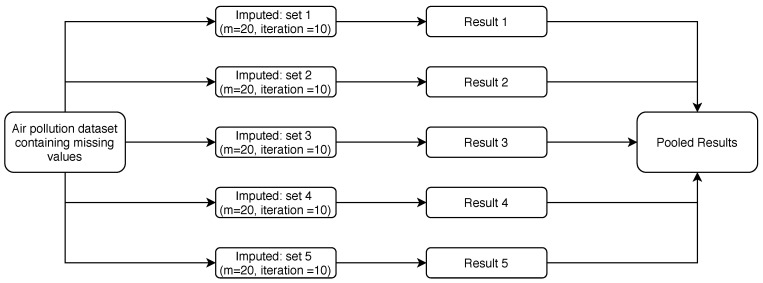
The steps of implementing multiple imputations for $PM_{10}$, $SO_{2}$, $O_{3}$, CO, and $NO_{2}$ during 2012 to 2017, according to site location, in the State of Kuwait.

For our data sets, we followed Rubin’s rules [11] for handling missing data. The process of multiple imputations (MIs) was conducted separately for each monitoring station (see Figure 1). The first step in multiple imputation is to create values (“imputes” or “$m_{i}$”), with 10 iterations for each “$m_{i}$” to be substituted for the missing data. In order to create imputed values, we need to identify a model (say, a linear regression) that allows us to create imputes based on other variables in the data set (predictor variables). As we need to do this multiple times, in order to produce multiple-imputed data sets, we identify a set of regression lines which are similar to each other.

Figure 1 shows the process for the KEPA data sets, to process and estimate missing values using imputation methods. There were five data sets (1–5), relating to FAH, JAH, MAN, RUM, and ASA, respectively. Each data set should contain 2192 daily observations for each variable; however, due to missing values, they were all less than 2192.

2 The power of MI lies in its multiple imputations being able to be performed for each variable in the data set. While every single imputation is ambiguous or imprecise, the combination of the computed imputations takes the uncertainty of each imputation into consideration. According to [17,18], MAR or MCAR pooled estimated parameters are less biased and the associated standard errors are corrected appropriately.

The implementation of an MI technique requires three steps: First, it imputes several values for the same observation, using at least two methods (m≥2). Then, the second step takes each individual method, *m*, and analyzes it using standard complete data. Finally, *m* (the completed data sets) is pooled by integrating the *m* analyses, in order to generate overall estimates and standard errors. This can be done by calculating the mean over the *m* repeated analyses. Pooling data from several *m* allows multiple imputations to ensure higher accuracy [30]. Figure 1 shows how we treated the KEPA data sets with multiple imputation, where m=20.

### 2.3. Data Sets

We utilized a real-time air quality monitoring data set collected for 5 locations in Kuwait from the Kuwait Environmental Public Authority (KEPA), in order to evaluate and assess the performance of various imputation methods to estimate missing values in the data set. The data set contained air quality, time, and meteorological data.

1. Air quality data: The air pollutant variables in the air quality data were $NO_{2}$, CO, $PM_{10}$, $SO_{2}$, and $O_{3}$;
2. Meteorological data: The meteorological parameters included temperature, humidity, wind direction, and wind speed.

All these variables for the past 24 h are collected on hourly basis and features extracted from the collected data set were used for evaluation of the models, for predictions of the concentration of missing values for $NO_{2}$, CO, $PM_{10}$, $SO_{2}$, and $O_{3}$. Concentrations of all the pollutants are reported in g/m${}^{3}$.

We compiled pollutant data from the Environmental Public Authority of Kuwait (KEPA). The data were gathered from five environmental monitoring stations from 1 January 2013 to 31 December 2017. We used the following pollutants: Particulate matter 10 micrometers ($PM_{10}$), nitrogen dioxide ($NO_{2}$), ozone ($O_{3}$), carbon monoxide (CO), and sulfur dioxide ($SO_{2}$). We estimated a concentration time of 24 h (daily observation) for $SO_{2}$, $NO_{2}$, and $PM_{10}$ at each station and 8 h for CO and $O_{3}$. We assumed 75% of the collected values as reliable averages [31]. We used the Air Quality Index (AQI), as generated by [32].

The AQI was developed, for Kuwait, based on the United States Environmental Protection Agency (USEPA) recommendations. The AQI is defined with consideration of characteristics of the air, in relation to the environmental needs of humans [32]. The AQI is an index for reporting the day-to-day air quality, providing details about the cleanliness of ambient air [33]. The following equation was used to convert between pollutant concentration to AQI:

(12) $$I_{p}=\frac{I_{high}-I_{low}}{C_{high}-C_{low}}\left(C_{p}-C_{low}\right)+I_{low},$$

where $I_{p}$ is the AQI for the given pollutant, $C_{p}$ is the pollutant concentration, $C_{low}$ is the concentration breakpoint that is $\leq C_{p}$, $C_{high}$ is the concentration breakpoint that is $\geq C_{p}$, $I_{low}$ is the index breakpoint corresponding to $C_{low}$, and $I_{high}$ is the index breakpoint corresponding to $C_{high}$ [34] (see Table 1).

Using the data obtained from KEPA, we conducted an in-depth comparative analysis of the different imputation methods. Missing data were entered into each data set, assuming a general missing data pattern and three mechanisms of missing data: MCAR, MAR, and NMAR. Under the MCAR assumption, missing values were randomly applied to each data set. Under the MAR assumption, the probability of information being missing depended on class attribute. Under the NMAR assumption, the largest or smallest values of $X_{s}$ were removed. The objective of the study was to derive a comparison of six different imputation methods for NMAR, MAR, and MCAR, concerning missing data. We simulated the rates of missing data by varying the value proportions by 5%, 10%, 20%, 30%, and 40%.

### 2.4. Evaluation Criteria

To determine the best imputation method, three model performance tests were considered [35]: root mean square error (RMSE), mean absolute error (MAE), and correlation coefficient (R), which are calculated as follows:

(13) $$RMSE=\sqrt{\frac{1}{n}\sum_{i=1}^{n}{\left(y_{i}-{\hat{y}}_{i}\right)}^{2}},$$

(14) $$MAE=\frac{1}{n}\sum_{i=1}^{n}\left|y_{i}-{\hat{y}}_{i}\right|,$$

where $y_{i}$ and ${\hat{y}}_{i}$ are the *i*th observations for the reconstructed and the comparison data sets, respectively. The error was measured based on the difference between the estimated value and the observed values. For RMSE and MAE tests, if the value obtained is small, then the estimation method is better.

### 2.5. R Packages Used for Imputation Process

Five well-known imputation packages accessible in R were applied. The first R package used here was VIM (https://cran.r-project.org/web/packages/VIM/VIM.pdf), which is associated with kNN imputation methods and robust model-based imputation for numerical, semi-continuous, categorical, or ordered variables [36]. The second R package was MICE (https://cran.r-project.org/web/packages/mice/mice.pdf) which stands for Multivariate Imputation via Chained Equations [37]. MICE is specialized to deal with missing values of MAR or MNAR types [38]. MICE can deal with different types of variables using different imputation methods, such as predictive mean matching for numeric variables, logistic regression for binary variables, Bayesian polytomous regression for factor variables, and a proportional odds model for ordered variables [38,39]. The third package was missForest (https://cran.r-project.org/web/packages/missForest/missForest.pdf). MissForest deals with non-parametric imputation [28]. MissForest enables the imputation of the predictors by using regression trees of resampling under the prediction classification of missing values [40]. MissForest has good computational efficiency and can work well with high-dimensional data [28]. The fourth package was Amelia (https://cran.r-project.org/web/packages/Amelia/Amelia.pdf), which enables imputation by maximizing the level of expectation with a bootstrapping algorithm. The Amelia package has also been recommended under a larger number of variables with high-dimensional data. The package also provides improved imputation models by adding Bayesian priors on individual cell values [41]. The final package used was missCompare (https://cran.r-project.org/web/packages/missCompare/missCompare.pdf). The missCompare package provides several diagnostic measurements to compare between all imputation methods, using RMSE, MAE, and other imputation performance criteria.

## 3. Statistical Results

Based on results for the real-time ambient air quality and meteorological data from the monitoring stations in KEPA, we inferred real-time and fine-grained ambient air quality information using means and standard deviations. The distribution analysis was conducted using the skewness and kurtosis with information of the quartiles (e.g., 25th and 75th quartiles, median, and &IQR&), where the correlation between the predictors was assessed by the Pearson correlation coefficient. The rate of missing values is presented for each monitoring station using the percentage of total number of missing values among the predictors.

**Figure A1:**
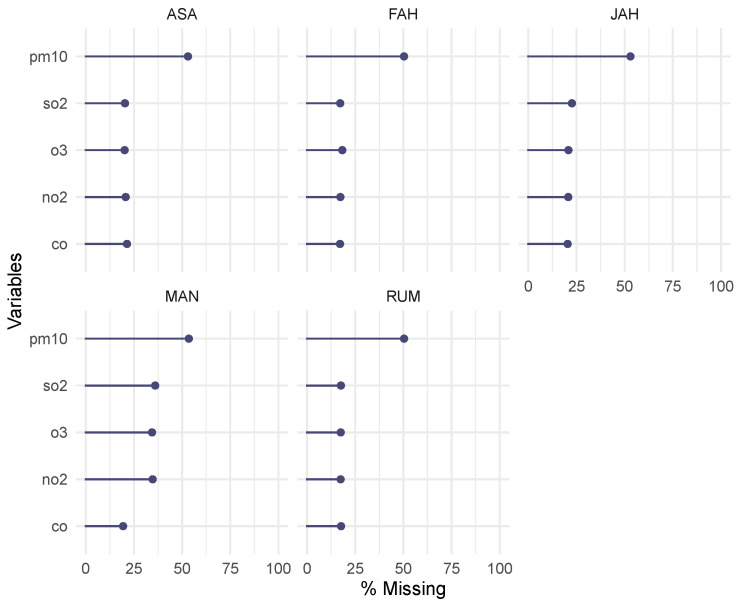
Missing values for air quality pollutants from 2012 to 2017 per fixed station.

**Figure A2:**
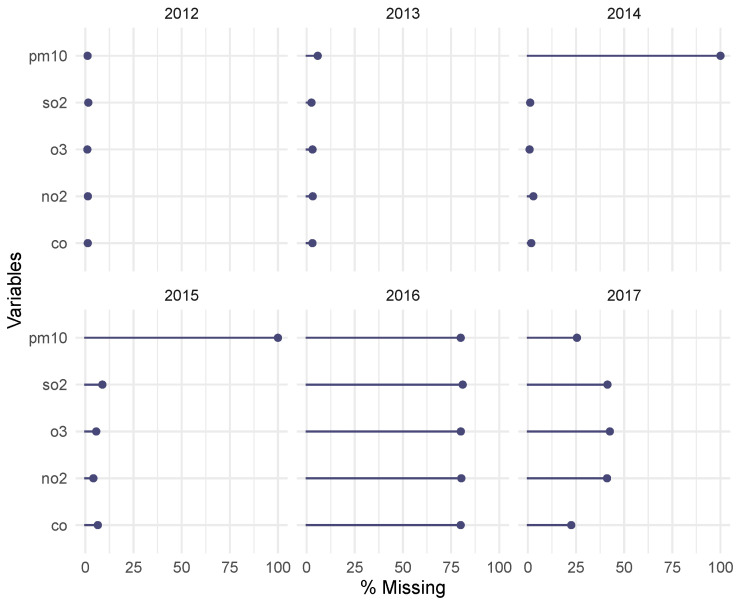
Missing values for air quality pollutants from 2012 to 2017 per year.

Table 2 shows the average air pollutant concentrations. The overall mean and SD for *PM*${}_{10}$, *CO*, *NO*${}_{2}$, *O*${}_{3}$, and *SO*${}_{2}$ were 0.23 ± 1.07, 0.91 ± 0.90, 0.04 ± 0.02, 0.02 ± 0.01, and 0.01 ± 0.01, respectively. The missing value rates were 52.16%, 19.37%, 22.35%, 22.40%, and 22.93% from all (N = 9006), respectively. Figure A1 and Figure A2 from Appendix A show the missing data distribution, based on year and monitoring site.

All pollutant distributions were positively skewed and we corrected the skewness by applying log transformations [31]. Figure A4 in the Appendix A shows the distribution performance after we applied logarithmic transformations to $PM_{10}$, $SO_{2}$, $O_{3}$, CO, and $NO_{2}$.

Table 3 shows the Pearson correlation analysis of various air pollutants and meteorological parameters. The strongest positive correlation was found between $NO_{2}$ and $SO_{2}$. This was expected, due to their common emission sources (e.g., road traffic). $NO_{2}$ had a weak association with $PM_{10}$, whereas $O_{3}$ had a highly negative association with $NO_{2}$. All meteorological parameters (temperature, humidity, wind speed, and wind direction) showed a negative association with $NO_{2}$.

**Figure 2:**
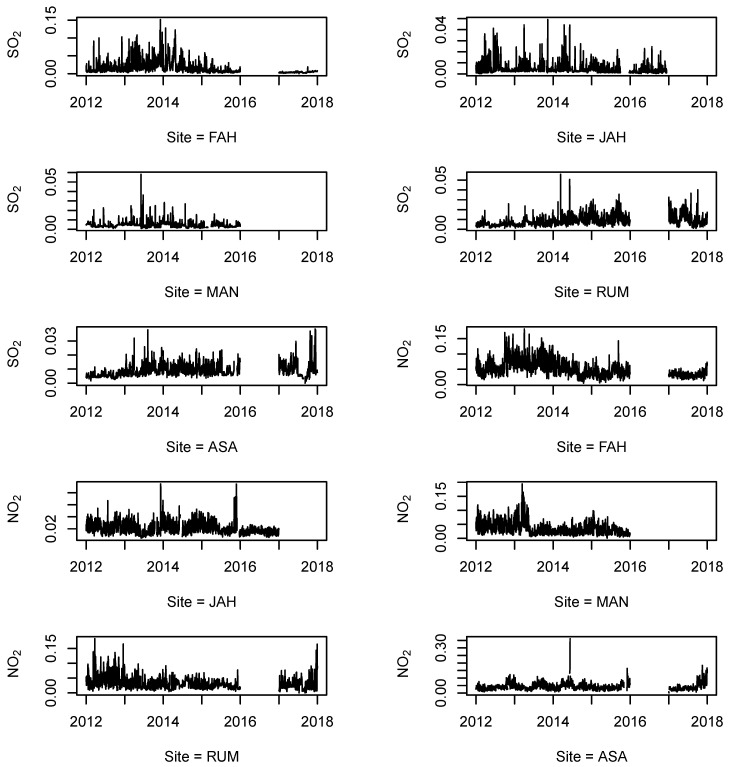
Time-series of air quality monitoring for $SO_{2}$ and $NO_{2}$ from 2012 to 2017, with missing values from five different locations (stations) in the State of Kuwait.

**Figure 3:**
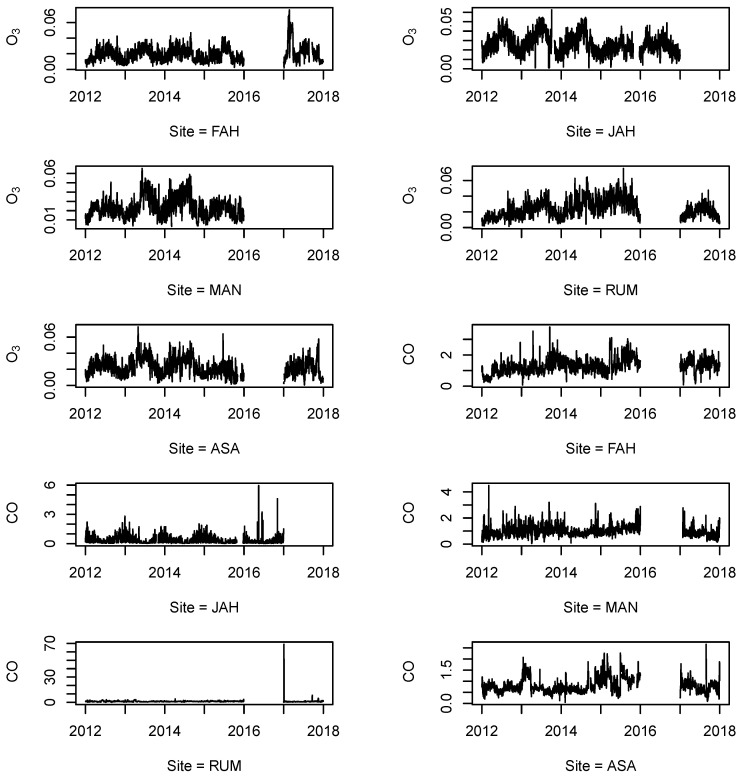
Time-series of air quality monitoring for $O_{2}$ and CO from 2012 to 2017, with missing values from five different locations (stations) in the State of Kuwait.

**Figure 4:**
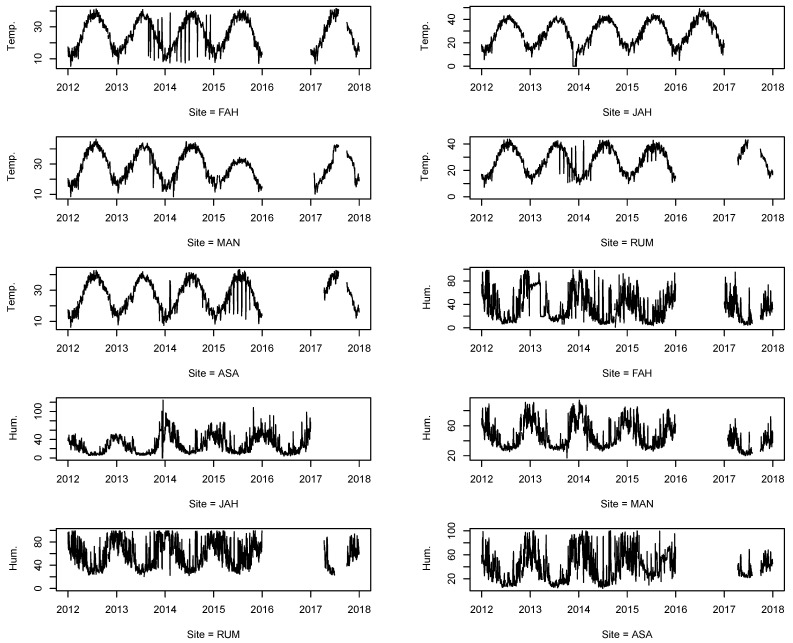
Time-series of weather climatology (temperature and relative humidity) from 2012 to 2017, with missing values from five different locations (stations) in the State of Kuwait.

**Figure A3:**
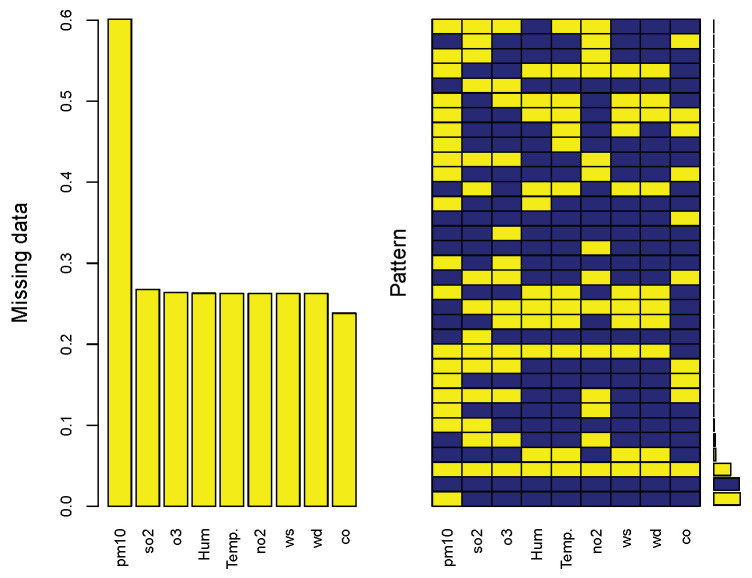
Missing value patterns for air quality measurements from 2012 to 2017. **Left**: Frequency of missingness in each variable. **Right**: Observed missingness patterns in the data set. The least frequent occurring patterns are located at the top of the plot, with gradually increasing frequency towards the bottom. Blue: observed, Yellow: missing.

**Figure A4:**
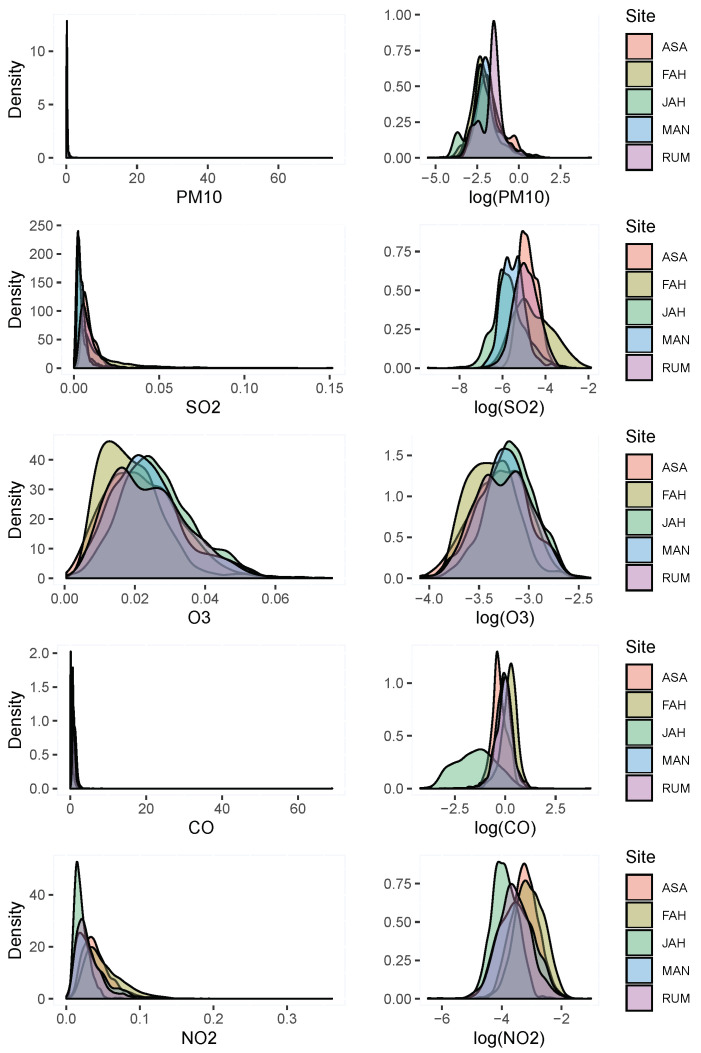
Distribution analysis for $PM_{10}$, $SO_{2}$, $O_{3}$, CO, and $NO_{2}$ during 2012 to 2017, according to site location in the State of Kuwait. It is very obvious that log transformation fixes the distribution shape for all pollutants. This step is very important—that is, normalizing the skewed data, such that they approximately conform to normality—in order to use them in the imputational calculation for more accurate results [56].

We performed time series plot for each pollutant for each monitoring station to better understand the patterns of the missing data among all observations (see Figure 2, Figure 3 and Figure 4). We concluded that the missing data pattern can be classified as missing at random (MAR) or missing not at random (MNAR), especially for the large missing gaps (see Appendix A, Figure A1, Figure A2, Figure A3). Figure A3 from Appendix A shows missing observation ratios for each pollutant. From Figure A3, we can conclude that PM10 has the highest missing observation rate among the pollutants (see Appendix A Figure A3-left panel). The right side of the Figure A3 from Appendix A shows the missing value pattern for each pollutant. The vertical connected blocks present the non-randomness for missing data during the monitoring.

Table 4 shows a comparison of missing rates for each monitored pollutant between monitoring stations. There were significant differences among the stations in producing missing values, where all *p*-values were less than 0.05, except for that of $PM_{10}$. $PM_{10}$ was excluded from all imputation calculations, due to a missing rate level that exceeded 50% [42,43].

### Missing Data Patterns

**Figure A5:**
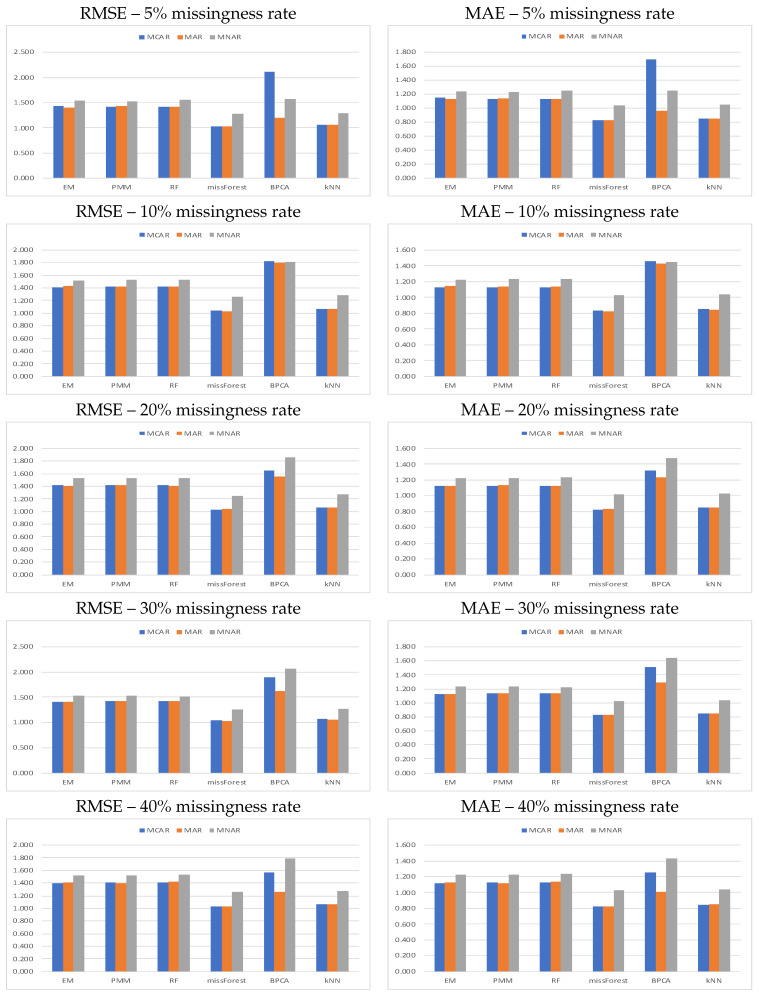
Mean RMSE and MAE results for the Kuwait Environmental Public Authority (KEPA) data, in order to estimate missing values for $SO_{2}$, $NO_{2}$, CO, and $O_{3}$ after eliminating $PM_{10}$ due to a high level of missing values. Results are shown for MCAR (**left**), MAR (**middle**), and MNAR (**right**) data.

As shown in Table 5 and Figure A5 from Appendix A, the RMSE ranged between 1.029 to 2.110 for MCAR, 1.028 to 1.431 for MAR, and 1.255 to 2.060 for MNAR; thus, MAR had the lowest rate of RMSE among the other missing data approaches. For MAR, the RMSE ranged between 0.821 to 1.145 for MCAR, 0.820 to 1.140 for MAR, and 1.019 to 1.478 for MNAR. This suggests that MAR had the lowest rate of MAR amongst the other missing pattern approaches. This result was consistent with previous studies [44,45]. As seen in Table 5 and appendix Figure A5, the best imputation method for estimating the simulated missing data was the missForest method. The missForest method had the smallest values of MAE and RMSE for all parameters and percentages of simulated missing data rates, this finding was consistent with the study of [1], where MTB was the best imputation method for filling the missing data, as it was able to obtain the smallest error for all percentages of missing data, in agreement with [28,44,46,47,48,49]. The second-best imputation method for estimating the simulated missing data was the k-nearest neighbor (kNN) method. This method performed better than the multiple imputation (MI) method for almost all parameters and proportions of missing data. This finding was consistent with the study reported by [42]. The worst-performing methods were multiple imputation using additive regression, bootstrapping, and predictive mean matching (PMM) methods. This was also consistent with the study reported by [42].

From Table 4, we can conclude that the missing rates are different among the selected air monitoring stations for each pollutant except $PM_{10}$ that shows similarities in missing rates among the monitoring stations. In addition, we can figure out from Appendix A Figure A3 how the missing values are distributed for each pollutant.

**Figure A6:**
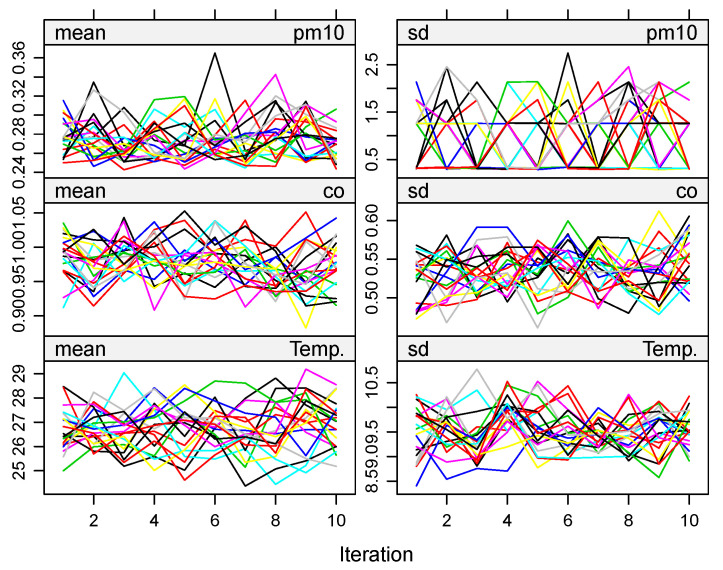
Inspecting the trace line convergence levels using an iterative Markov Chain–Monte Carlo type of algorithm with respect to the imputed means and standard deviations for $PM_{10}$, CO, and temperature. These trace plots show the imputed value summaries for all imputed data sets with m=20 after applying 10 iterations, in order to reach to the convergence level of stability.

**Figure A7:**
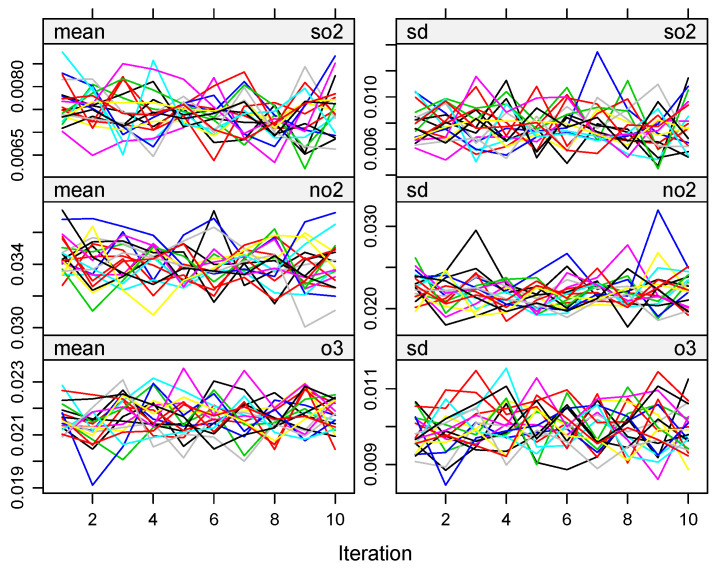
Inspecting the trace line convergence levels using an iterative Markov Chain–Monte Carlo type of algorithm with respect to the imputed means and standard deviations for $SO_{2}$, $NO_{2}$, and $O_{3}$. These trace plots show the imputed value summaries for all imputed data sets with m=20 after applying 10 iterations, in order to reach to the convergence level of stability. Each color in the graph represents an imputed data set, where the x-axis represents the number of iterations implemented during the imputational calculation and the y-axis represents the mean (**left**-side) and standard deviation (**right**-side) of the imputed values only.

**Figure A8:**
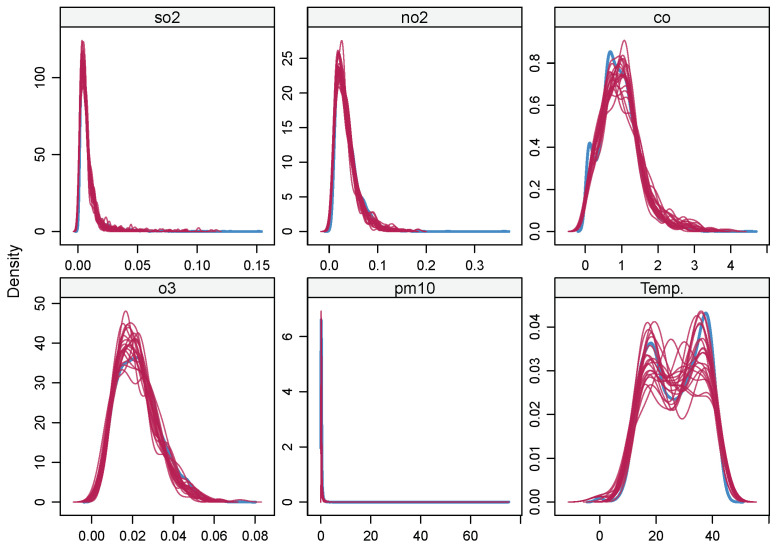
Density plots with multiple imputations for $SO_{2}$, $NO_{2}$, $PM_{10}$, CO, and $O_{3}$ data. The blue line represents the observed data and the red lines are the density plots of the 20 imputed data sets. As we can see, in all density plots, the red lines almost match the blue line (the observed data), which is an indication of matching between the observed and imputed values.

The results of the missing imputation approach were diagnosed using convergent plots for the mean and standard deviation of the multiple imputation data sets using missForest (see Appendix A Figure A6 and Figure A7). For convergence, the different streams should not show any definite trends; we did not observe any obvious trends in these data. In addition, Figure A8 shows Kernel density estimates for the marginal distributions of the observed data (blue line) and the m=20 densities per variable calculated from the imputed data (red lines). This indicates stability after 10 iterations.

**Figure 5:**
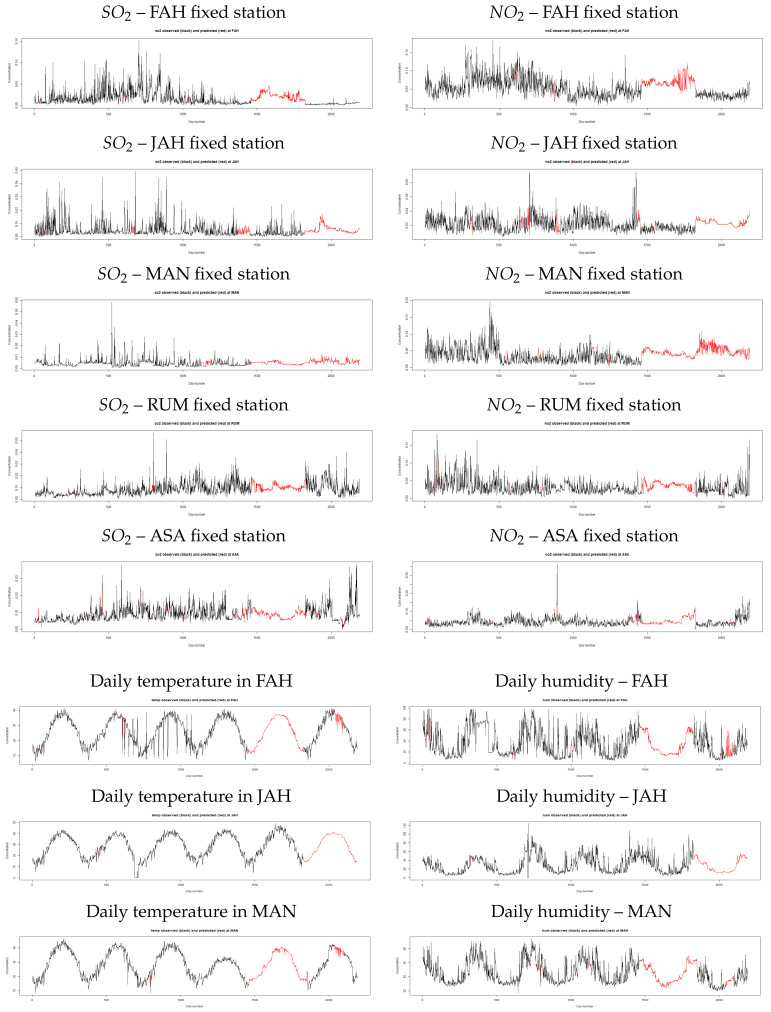
Daily concentrations of $SO_{2}$, $NO_{2}$, temperature, and relative humidity after estimating missing values using the missForest approach (from 2012–2017).

**Figure 6:**
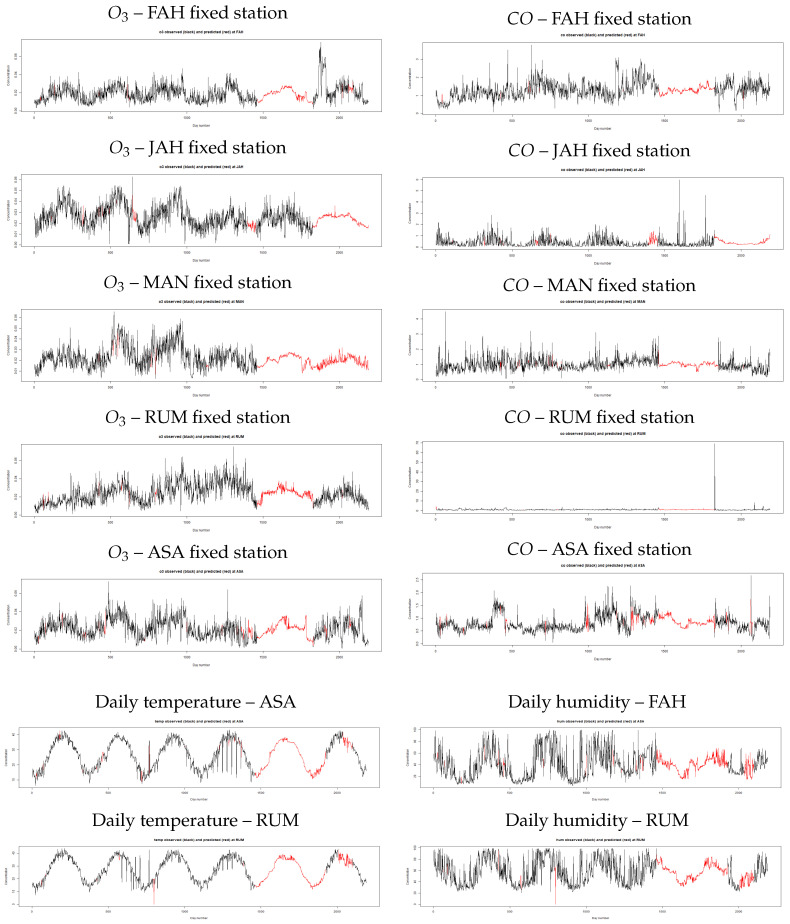
Daily concentrations of $O_{3}$, CO, temperature, and relative humidity after estimating missing values using the missForest approach (from 2012–2017).

We imputed the missing information into the original data sets to assess if the imputed data are consistent with the existing data. Figure 5 and Figure 6 showed how imputed datasets fit with the actual information in each station. We can see from the figures that large gaps of missing data are filled in the same pattern of the historical values for all pollutants and meteorological parameters which gives a good indication of using missForest to estimate missing air pollutants.

## 4. Discussion

In Kuwait, the Environmental Public Authority (KEPA) is responsible for monitoring the air quality status. The data of air quality obtained from the five stations used in this study usually contain missing data, which can cause bias due to systematic errors between the observed and unobserved values [31]. Therefore, it is vital to determine the optimal approach for estimating the missing values, in order to guarantee that the analyzed data are of high quality. Incomplete data matrices may provide outcomes that vary significantly, compared to the results expected from a data set that is complete [4]. The primary purpose of any data analysis is to make valid and reasonable inferences on a particular population under study. A researcher is expected to respond to the missing data problem in a way that aligns with the population of interest.

There have been many contributions to this field, such as in environmental [1,7,50,51], statistical [52,53], and medical studies [54,55]. In the environmental field, imputation is the statistical procedure of assigning inferential values to recover all missing data using prior knowledge from other predictors.

The existence of efficient imputation algorithms has led to the extensive usage of elaborate imputation methods across the world. As more people become knowledgeable about imputation algorithms, inquisitiveness regarding the methodology increases, leading to the invention of more sophisticated imputation methods. However, the main challenge concerning imputed values is whether to consider them as actual measurements or to be handled with caution. In the field of research, it is preferable to handle assigned figures with great discretion. This is because the use of imputed figures as actual data may lead to a misguided impression, which may potentially falsify the final results. Therefore, the imputed values should be given low priority.

It is, therefore, vital for a researcher to impute missing data and assess how robust the associated data estimation is. Environmental information that relies on technological processing and simulation poses a challenge. Missing data ascription is one approach: A substantial quality of ascription methods is that they are reliable and limited to one type of variable. This variable may be considered as persistent or unmitigated. If the data type is blended, the method must deal with the different types of data separately. In conclusion, these techniques ignore the potential associations between different factor types. For the situation here, before conducting any statistical modeling or performing time-series analysis, it is better to treat the missing values and to try to estimate them using other information from other predictors. This may help to avoid any bias circumstance and to enhance model performance for better estimation.

The main contribution of this paper was to find the most appropriate method to fill in missing observations in an air pollution data set from Kuwait. Single and multiple imputation methods were adopted and their performances were compared using using the RMSE and MAE metrics. To estimate missing data for $SO_{2}$, $NO_{2}$, $PM_{10}$, CO, and $O_{3}$ in the KEPA database, we applied artificially introduced missing values ranging from 10% to 40%. We showed that missForest could successfully handle the missing values, particularly in data sets including different types of environmental variables.

However, this computation method also had limitations. It requires proficiency in R programming, being demanding in comparison to the kNN or PMM methods. There is also a possible connection between the pollutant values and the missing variables. Therefore, these results are not applicable in cases where the missing data are due to non-random reasons. It is evident that some of the observed air pollutant records contained erroneous information. When we ignore this factor during the examination, the results obtained tend to be misleading.

Our findings revealed that missForest was the only imputation method with a consistent and comparatively lower imputation error (of 0.82). The approach had a root mean square error of 1.04. missForest also exhibited the smallest prediction deviation in the imputed values of pollutants. Furthermore, missForest simulation provides the most readily available imputation of missing values, as its freeware R package is freely available.

While compiling the report of the study, we assumed the missing at random (MAR) tool. This premise is essential for the development of a prototype of the observation for the imputation of missing data. There was a possibility of the missing data system being not missing at random (NMAR). In such a case, the missing variables are directly related to their causes. It may be challenging to determine the actual missing data mechanism, in such a case. Therefore, distinguishing between NMAR and MAR would involve a thorough investigation of the data capturing process. Other assumptions include Gaussian-distributed data, which may have been erroneous for some variables. Using the appropriate distribution for each variable can help to reduce this error. This might increase the reliability of the MICE imputation results, which determine the mechanism for each variable.

## 5. Conclusions

Missing data are always lost, in their entirety and forever, but a proper imputation scheme can help to remedy the situation as much as possible. The method that performs best in each situation, in terms of the assessments, is made in this work. For this study, missForest gives the most accurate results in estimating the missing values through the multi-dimensional dataset (the datasets that came from five fixed monitoring stations). The missForest method enables imputation on virtually any kind of data. In particular, it can deal with multivariate information comprised of continuous and categorical factors at the same time. This method does not require parameter tuning, nor does it require assumptions about the distribution of the information. Finally, missForest had the least imputation error for both continuous and categorical variables at each frequency of missingness rates (5%, 10%, 20%, 30%, and 40%), and it had the smallest prediction error difference when models used imputed values.

## Figures and Tables

**Table 1:** Kuwait Air Quality Index.

Categories	AQI Sub-Index	O${}_{3}$ (ppm) 8-h	PM${}_{10}$ (µg/m${}^{3}$) 24-h	CO (ppm) 24-h	SO${}_{2}$ (ppm) 24-h	NO${}_{2}$ (ppm) 24-h
	$I_{\mathrm{low}}$–$I_{\mathrm{high}}$	$I_{\mathrm{low}}$–$I_{\mathrm{high}}$	$I_{\mathrm{low}}$–$I_{\mathrm{high}}$	$I_{\mathrm{low}}$–$I_{\mathrm{high}}$	$I_{\mathrm{low}}$–$I_{\mathrm{high}}$	$I_{\mathrm{low}}$–$I_{\mathrm{high}}$
Good	0–50	0.0–0.03	0.0–90	0.0–4.0	0.0–0.03	0.0–0.03
Moderate	51–100	0.031–0.06	90.1–350.0	4.1–8.0	0.031–0.06	0.04–0.05
Unhealthy (1)	101–150	0.061–0.092	350.1–431.1	8.1–11.7	0.061–0.182	0.06–0.30
Unhealthy (2)	151–200	0.093–0.124	431.4–512.5	11.8–15.4	0.183–0.304	0.31–0.55
Very Unhealthy	201–300	0.125–0.374	512.6–675.0	15.5–30.4	0.305–0.604	0.56–1.04
Hazardous	301–500	0.375–0.504	675.1–1000	30.5–50.4	0.605–1.004	1.05–2.04

**Table 2:** Distribution of Kuwait ambient air pollution exposure during 2012–2017. The total daily observations for ASA are N = 1779; for FAH, N = 1820; for JAH, N = 1819; for MAN, N = 1777; and, for RUM, N = 1811.

Air Pollutant	ASA	FAH	JAH	MAN	RUM	All (N = 9006)
${PM}_{10}$						
min	0.017	0.004	0.005	0.008	0.019	0.004
25th	0.099	0.076	0.073	0.099	0.121	0.088
median	0.154	0.109	0.107	0.142	0.211	0.140
75th	0.262	0.163	0.180	0.218	0.273	0.232
max	3.248	5.500	1.714	7.216	2.538	7.216
mean (sd)	0.26 ± 0.32	0.17 ± 0.28	0.17 ± 0.20	0.32 ± 2.38	0.25 ± 0.23	0.23 ± 1.07
%Missing	%53.16	%50.43	%53.12	%53.62	%50.48	%52.16
CO						
min	0.050	0.078	0.015	0.048	0.015	0.015
25th	0.597	0.981	0.107	0.719	0.743	0.562
median	0.720	1.265	0.235	0.922	0.971	0.860
75th	0.945	1.567	0.471	1.172	1.241	1.198
max	2.661	3.789	5.956	4.483	68.980	68.980
mean (sd)	0.80 ± 0.32	1.30 ± 0.47	0.36 ± 0.41	0.98 ± 0.41	1.08 ± 1.68	0.91 ± 0.90
%Missing	%21.57	%17.30	%20.57	%19.57	%17.84	%19.37
${NO}_{2}$						
min	0.001	0.005	0.004	0.001	0.000	0.000
25th	0.028	0.032	0.014	0.018	0.018	0.020
median	0.038	0.045	0.019	0.029	0.026	0.030
75th	0.052	0.066	0.026	0.046	0.039	0.046
max	0.361	0.182	0.095	0.194	0.183	0.361
mean (sd)	0.04 ± 0.02	0.05 ± 0.03	0.02 ± 0.01	0.03 ± 0.02	0.03 ± 0.02	0.04 ± 0.02
%Missing	%20.89	%17.48	%20.89	%34.87	%17.61	%22.35
$O_{3}$						
min	0.001	0.002	0.001	0.003	0.001	0.001
25th	0.014	0.012	0.019	0.017	0.015	0.015
median	0.021	0.018	0.025	0.022	0.023	0.022
75th	0.029	0.024	0.033	0.029	0.031	0.029
max	0.073	0.076	0.062	0.065	0.075	0.076
mean (sd)	0.02 ± 0.01	0.02 ± 0.01	0.03 ± 0.01	0.02 ± 0.01	0.02 ± 0.01	0.02 ± 0.01
%Missing	%20.35	%18.48	%20.98	%34.55	%17.66	%22.40
${SO}_{2}$						
min	0.000	0.000	0.000	0.001	0.001	0.000
25th	0.006	0.005	0.002	0.003	0.005	0.004
median	0.008	0.009	0.003	0.004	0.007	0.006
75th	0.011	0.019	0.005	0.005	0.011	0.010
max	0.038	0.152	0.049	0.058	0.056	0.152
mean (sd)	0.01 ± 0.00	0.02 ± 0.02	0.00 ± 0.00	0.00 ± 0.00	0.01 ± 0.01	0.01 ± 0.01
%Missing	%20.53	%17.39	%22.80	%36.19	%17.75	%22.93

**Table 3:** Correlation analysis between weather climatology and air-pollution components $SO_{2}$, $NO_{2}$, $O_{3}$, CO, and $PM_{10}$.

	${NO}_{2}$	$O_{3}$	${SO}_{2}$	CO	${PM}_{10}$	Temp.	Hum.	Wind Speed
$NO_{2}$								
$O_{3}$	−0.35 ***							
$SO_{2}$	0.40 ***	−0.09 ***						
CO	0.35 ***	−0.26 ***	0.22 ***					
$PM_{10}$	−0.06 ***	0.05 **	−0.03 *	−0.03				
Temp.	−0.09 ***	0.45 ***	−0.06 ***	−0.14 ***	0.05 **			
Hum	−0.02	−0.25 ***	−0.08 ***	0.29 ***	−0.03	−0.61 ***		
Wind Speed	−0.20 ***	0.30 ***	0.13 ***	−0.22 ***	0.10 ***	0.24 ***	−0.32 ***	
Wind Direction	−0.25 ***	0.13 ***	−0.15***	−0.27 ***	0.06 ***	0.14 ***	−0.28 ***	0.31 ***

Note: * *p* < 0.1; ** *p* < 0.05; *** *p* < 0.01; **** *p* < 0.001.

**Table 4:** Missing data by site. From the results we conclude that all monitoring fixed stations are different in missing values amount for each pollutant.

	ASA	FAH	JAH	MAN	RUM	*p*-Value
	N = 2192	N = 2192	N = 2192	N = 2192	N = 2192	
$NO_{2}$	454 (20.7%)	379 (17.3%)	454 (20.7%)	761 (34.7%)	382 (17.4%)	<0.001
$O_{3}$	442 (20.2%)	401 (18.3%)	456 (20.8%)	754 (34.4%)	383 (17.5%)	<0.001
$SO_{2}$	446 (20.3%)	377 (17.2%)	496 (22.6%)	790 (36.0%)	385 (17.6%)	<0.001
CO	469 (21.4%)	375 (17.1%)	447 (20.4%)	425 (19.4%)	387 (17.7%)	0.001
$PM_{10}$	1163 (53.1%)	1103 (50.3%)	1162 (53.0%)	1173 (53.5%)	1104 (50.4%)	0.069

**Table 5:** RMSE comparison between the indexed original values and the imputed values using missing at random (MAR), missing completely at random (MCAR) and missing not at random (MNAR) missingness patterns. From the results, it is very obvious that the MAR technique has the lowest RMSE scores among the other techniques. We can also see that missForest had the lowest RMSE and MAE, among the other imputation methods, for all missing rate criteria.

Method	5% Missingness Rate					
	RMSE			MAE		
	MCAR	MAR	MNAR	MCAR	MAR	MNAR
EM	1.430	1.405	1.536	1.145	1.120	1.238
PMM	1.408	1.430	1.529	1.129	1.140	1.225
RF	1.413	1.412	1.547	1.128	1.126	1.242
missForest	1.031	1.035	1.270	0.821	0.823	1.036
BPCA	2.110	1.199	1.568	1.686	0.953	1.251
kNN	1.064	1.065	1.288	0.850	0.846	1.047
**Method**	**10% missingness rate**					
	**RMSE**			**MAE**		
	**MCAR**	**MAR**	**MNAR**	**MCAR**	**MAR**	**MNAR**
EM	1.408	1.431	1.517	1.125	1.140	1.218
PMM	1.414	1.415	1.527	1.125	1.131	1.229
RF	1.414	1.416	1.529	1.129	1.133	1.231
missForest	1.035	1.028	1.260	0.829	0.820	1.025
BPCA	1.816	1.792	1.813	1.456	1.431	1.449
kNN	1.063	1.064	1.282	0.853	0.846	1.041
**Method**	**20% missingness rate**					
	**RMSE**			**MAE**		
	**MCAR**	**MAR**	**MNAR**	**MCAR**	**MAR**	**MNAR**
EM	1.415	1.410	1.523	1.129	1.124	1.225
PMM	1.418	1.417	1.528	1.129	1.131	1.226
RF	1.413	1.408	1.532	1.128	1.124	1.228
missForest	1.029	1.038	1.253	0.819	0.827	1.019
BPCA	1.653	1.548	1.856	1.319	1.233	1.478
kNN	1.062	1.065	1.270	0.847	0.850	1.032
**Method**	**30% missingness rate**					
	**RMSE**			**MAE**		
	**MCAR**	**MAR**	**MNAR**	**MCAR**	**MAR**	**MNAR**
EM	1.405	1.410	1.531	1.124	1.127	1.232
PMM	1.418	1.419	1.527	1.131	1.132	1.229
RF	1.419	1.419	1.521	1.136	1.134	1.224
missForest	1.034	1.033	1.255	0.825	0.823	1.023
BPCA	1.891	1.622	2.060	1.506	1.293	1.645
kNN	1.065	1.064	1.276	0.850	0.848	1.036
**Method**	**40% missingness rate**					
	**RMSE**			**MAE**		
	**MCAR**	**MAR**	**MNAR**	**MCAR**	**MAR**	**MNAR**
EM	1.401	1.411	1.518	1.119	1.127	1.222
PMM	1.411	1.399	1.520	1.126	1.116	1.222
RF	1.412	1.419	1.534	1.124	1.133	1.234
missForest	1.032	1.035	1.259	0.823	0.827	1.027
BPCA	1.564	1.264	1.789	1.250	1.007	1.428
kNN	1.062	1.067	1.279	0.847	0.852	1.042

## Data Availability

Not applicable.

## Appendix B. Algorithms-MissForest

**Algorithm A1:** Impute missing values with random forest [28].
**Require:** **X** is an *n* × *p* matrix, setup stopping criterion (γ) setup initial guess for missing values; k← vector of sorted indices of columns in X w.r.t. increasing amount of missing values; **while** not γ **do** $X_{old}^{imp}\leftarrow$ store previously imputed matrix; **for** *s* in k **do** Fit a random forest: $y_{obs}^{\left(s\right)}∼x_{obs}^{\left(s\right)}$; Predict $y_{mis}^{\left(s\right)}$ using $x_{mis}^{\left(s\right)}$; $X_{new}^{imp}\leftarrow$ update imputed matrix, using predicted $y_{mis}^{\left(s\right)}$; **end for** update γ **end while** **return** the imputed matrix $X^{imp}$
